# Prognostic values of tissue factor and its alternatively splice transcripts in human gastric cancer tissues

**DOI:** 10.18632/oncotarget.17942

**Published:** 2017-05-16

**Authors:** Min Wu, Lujun Chen, Ting Xu, Bin Xu, Jingting Jiang, Changping Wu

**Affiliations:** ^1^ Department of Tumor Biological Treatment, The Third Affiliated Hospital of Soochow University, Jiangsu Changzhou 213003, China; ^2^ Research Center for Cancer Immunotherapy of Jiangsu Province, The Third Affiliated Hospital of Soochow University, Jiangsu Changzhou 213003, China; ^3^ Department of Rheumatology, The Third Affiliated Hospital of Soochow University, Jiangsu Changzhou 213003, China; ^4^ Institute of Cell Therapy, The Third Affiliated Hospital of Soochow University, Jiangsu Changzhou 213003, China; ^5^ Department of Oncology, The Third Affiliated Hospital of Soochow University, Jiangsu Changzhou 213003, China

**Keywords:** gastric cancer, tissue factor, alternative splice, prognosis

## Abstract

We have previously reported that the higher expression of TF in human esophageal cancer tissues was significantly associated with tumor invasion, intratumoral microvessel density and patients’ postoperative prognoses. Besides its trans-membranous form, TF also has alternatively spliced transcripts. In the present study, the transcripts of the two TF isoforms, flTF and asTF, in human gastric cancer tissues were determined by real-time PCR, and the correlation between the expression of TF isoforms and patient's clinicopathological features was also analyzed. Our results showed that the relative mRNA expression levels of flTF and asTF in human gastric cancer tissues was significantly higher than those in normal tissues (*P*=0.035 and *P*=0.006, respectively). The relative mRNA expression level of asTF was significantly associated with age (*P*=0.018), meanwhile, we could not find that flTF or asTF expression level was correlated with any other characteristics of the patients, including gender, TNM stage, pathological grade, tumor size, histological type, or chemotherapy sensitivity. Univariate analysis demonstrated that the overall survival rate of gastric cancer patients with lower flTF or asTF expression level was greater than those with higher expression level (*P*=0.018 and =0.038, respectively). Multivariate COX model analysis also demonstrated that flTF expression (*P*=0.048) or asTF expression (*P*=0.002) could be used as independent prognostic predictors in human gastric cancer. Thus, both flTF and asTF mRNA expression levels in cancer tissues could be used as useful risk factors for evaluating the prognoses of patients suffering from gastric cancer.

## INTRODUCTION

Gastric cancer is an important cancer type occurring in the upper digestive tract, and presents with high morbidity and mortality in China [1]. The extensive heterogeneity of this malignancy complicates the precise assessment of tumor aggressiveness and prognosis, complicating implementation of effective therapeutic strategies [2]. Thus, it is important to investigate the molecular mechanisms involved in the transformation and progression of gastric cancer. Additionally, there is a need to identify prognostic predictors and novel biomarkers that could predict certain patients benefitting from the targeted therapies.

Tissue factor (TF), a 47-kDa trans-membrane glycoprotein, is a cellular receptor for coagulation factor VII (FVII), activating a clotting cascade involved in many physio-pathological processes [3, 4]. It has been demonstrated that TF could be constitutively expressed by various extra-vascular cells and cancer cells [5]. TF could influence protease-activated receptor-dependent tumor cell behavior and regulate integrin function, leading to the intratumoral angiogenesis both *in vitro* and *in vivo* [6]. Our previous study demonstrated that the higher expression of TF in human esophageal cancer tissues significantly associated with tumor invasion and intratumoral angiogenesis, suggesting a positive role of TF in cancer progression [3]. Aberrant TF expression could be induced by a majority of oncogenic events, such as activation of K-Ras or epidermal growth factor receptor (EGFR), inactivation of p53 tumor suppressor, and loss of phosphatase and tensin homolog (PTEN) [7–9].

**Figure 1:**
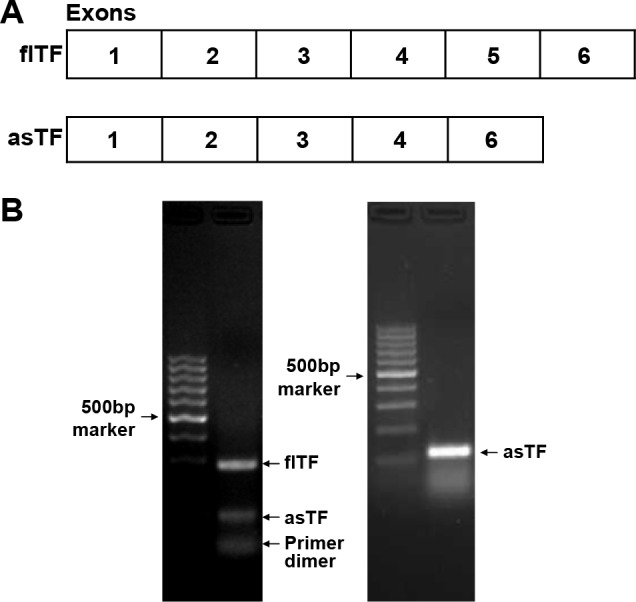
The structure of flTF and asTF **(A)** The exons of flTF and asTF. **(B)** The electrophoresis analysis of the PCR products of flTF and asTF. Left panel, the primers for flTF as well as asTF adopted from the reference. Right panel, the primers designed by ourselves which were used in the real-time PCR assay.

Besides its transmembrane form, TF also exhibits alternatively spliced transcripts. In 2003, Bogdanov *et al*. reported a novel alternatively-spliced human TF (*asTF*) in which exon 5 is deleted (as shown in Figure 1) [10]. The full-length TF (*flTF*) has six exons, and the *asTF* lacks exon 5, leading to a truncation of the transmembrane domain and a soluble form of TF [10]. Although lacking pro-coagulant activity, asTF could promote primary growth of human pancreatic cancer cells *in vivo* and augment tumor-associated angiogenesis [11, 12]. In this study, the transcripts of the two TF isoforms, flTF and asTF, in human gastric cancer tissues were assessed. These two transcripts in gastric cancer tissues and adjacent normal tissues were determined by real-time PCR. The correlation between the mRNA expression levels of TF isoforms and patient's clinicopathological features was also analyzed.

## RESULTS

### mRNA expression levels of flTF and asTF in human gastric cancer tissues and adjacent normal tissues

**Figure 2:**
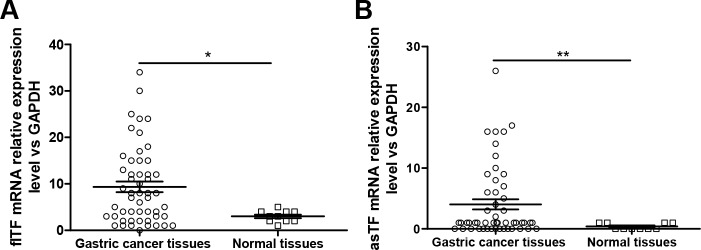
The mRNA expression levels of flTF and asTF in human gastric tissues **(A)** The mRNA expression level of flTF in gastric cancer tissues was significantly higher than that in normal gastric tissues. **(B)** The mRNA expression level of asTF in gastric cancer tissues was significantly higher than that in normal gastric tissues.

The relative mRNA expression level of flTF in human gastric cancer tissues [7.45 (0.34∼33.68)] was significantly higher than in normal control tissues [3.00 (1.36∼5.02)] (Figure 2A, *P*=0.035). In addition, the relative mRNA expression level of asTF in human gastric cancer tissues [0.88 (0.07∼26.00)] was also found significantly higher than in normal control tissues [0.33 (0.03∼0.97)] (Figure 2B, *P*=0.006). To determine the association of flTF and asTF expression in gastric cancer, we further sub-grouped the 52 patients into flTF^Low^ group (n=23), flTF^High^ group (n=29), asTF^Low^ group (n=31), or asTF^High^ group (n=20) based on the selected and relative cut-off levels for flTF and asTF (5.02 and 0.97, respectively).

### Relationship between clinical pathological variables and mRNA relative expression levels of flTF and asTF in gastric cancer tissues

In this study, the mRNA expression level of flTF was not associated with any patients’ clinical parameters (Table 1). The mRNA expression level of asTF in gastric cancer tissues was significantly associated with patients’ age (*P*=0.018), while it was not correlated with any other clinical parameters of the patients (Table 1).

**Table 1:** Correlation between patients’ clinical parameters and the mRNA expression levels of flTF and asTF in human gastric cancer tissues

Clinical parameters	Cases	flTF expression level		*χ^2^*	*P*-value	Cases	asTF expression level		*χ^2^*	*P-*value
		High (n, %)	Low (n, %)				High (n, %)	Low (n, %)		
Gender										
Male	34	18(52.9)	16(47.1)	0.318	0.573	34	15(44.1)	19(55.9)	1.028	0.311
Female	18	11(61.1)	7(38.9)			17	5(29.4)	12(70.6)		
Age (years)										
≤60	19	13(68.4)	6(31.6)	1.943	0.163	18	11(61.1)	7(38.9)	5.595	**0.018**
>60	33	16(48.5)	17(51.5)			33	9(27.3)	24(72.7)		
Tumor stage										
T_2_	3	2(66.7)	1(33.3)	2.484	0.289	3	1(33.3)	2(66.7)	1.106	0.575
T_3_	7	2(28.6)	5(71.4)			7	4(57.1)	3(42.9)		
T_4_	42	25(59.5)	17(40.5)			41	15(36.6)	26(63.4)		
Nodal stage										
N0	3	2(66.7)	1(33.3)	1.44	0.696	2	1(50.0)	1(50.0)	0.952	0.813
N1	8	3(37.5)	5(62.5)			8	2(25.0)	6(75.0)		
N2	18	11(61.1)	7(38.9)			18	7(38.9)	11(61.1)		
N3	23	13(56.5)	10(43.5)			23	10(43.5)	13(56.5)		
Distant metastasis										
No	45	25(55.6)	20(44.4)	0.006	0.937	44	17(38.6)	27(61.4)	0.045	0.832
Yes	7	4(57.1)	3(42.9)			7	3(42.9)	4(57.1)		
TNM stage										
I	1	1(100.0)	0(0.0)	2.092	0.553	1	0(0.0)	1(100.0)	0.686	0.876
II	6	2(33.3)	4(66.7)			5	2(40.0)	3(60.0)		
III	38	22(57.9)	16(42.1)			38	15(39.5)	23(60.5)		
IV	7	4(57.1)	3(42.9)			7	3(42.9)	4(57.1)		
Tumor size (cm)										
≤5	18	10(55.6)	8(44.4)	0.029	0.864	18	6(33.3)	12(66.7)	0.213	0.644
>5	31	18(58.1)	13(41.9)			30	12(40.0)	18(60.0)		
Invasion to the whole layer										
No	10	4(40.0)	6(60.0)	1.248	0.264	10	5(50.0)	5(50.0)	0.607	0.436
Yes	42	25(59.5)	17(40.5)			41	15(36.6)	26(63.4)		
Histological type										
Well-differentiated	28	12(42.9)	16(57.1)	3.808	0.051	28	7(25.0)	21(75.0)	2.316	0.128
Poor-differentiated	18	13(72.2)	5(27.8)			17	8(47.1)	9(52.9)		
Chemo-sensitivity										
Insensitive	19	12(63.2)	7(36.8)	0.012	0.912	18	6(33.3)	12(66.7)	0.121	0.728
Sensitive	26	16(61.5)	10(38.5)			26	10(38.5)	16(61.5)		

Values in bold signify *P* < 0.05.

### Prognostic values of mRNA expression levels of flTF and asTF in gastric cancer tissues

**Figure 3:**
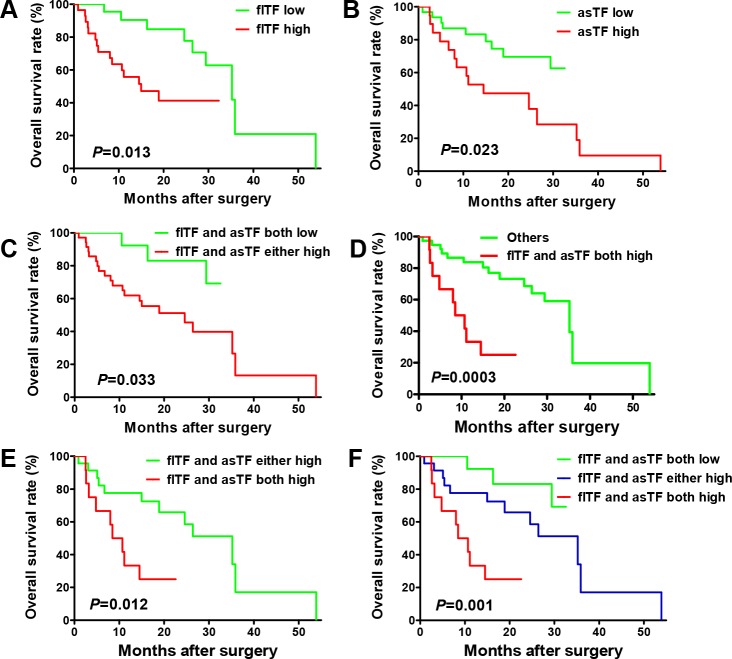
Prognostic value of flTF and asTF levels in human gastric cancer tissues **(A)** The overall survival rate of the patients with low flTF was significantly better than those with high flTF. **(B)** The overall survival rate of the patients with low asTF was significantly better than those with high asTF. **(C)** The overall survival rate of the patients with both low flTF and asTF was significantly better than those with flTF and asTF either high group. **(D)** The overall survival rate of the patients with flTF and asTF both high was significantly poorer than other patients. **(E)** The overall survival rate of the patients with flTF and asTF either high group was significantly better than those with flTF and asTF both high group. **(F)** There was a significant difference among the three subgroups, namely the patients with flTF and asTF both low, the patients with flTF and asTF either high, and the patients with flTF and asTF both high.

To determine whether flTF and asTF are prognostic factors in gastric cancer, we further carried out survival analyses based on the sub-groups stratified by low/high flTF and asTF expression. As shown in Table 2, we found that the patient's age (*P*=0.014), tumor size (*P*=0.009), and flTF expression level (*P*=0.018) was significantly associated with post-operative survival. COX model analyses showed that tumor size (*P*=0.009), distant metastasis (*P*=0.008), and flTF expression level (*P*=0.048) could be independent risk factors. The expression level of asTF was also significantly associated with patient's post-operative survival (Table 3, *P*=0.038). COX model analyses showed that histo-pathological type (*P*=0.012), tumor size (*P*=0.002), tumor differentiation (*P*=0.041), distant metastasis (*P*=0.005), and asTF expression level (*P*=0.002) were independent risk factors of gastric cancer. Also, as shown in Figure 3, we also found that the overall survival rate of the patients with both low flTF and asTF was significantly better than those with flTF and asTF either high group (Figure 3C, *P*=0.033). The overall survival rate of the patients with flTF and asTF both high was significantly poorer than other patients (Figure 3D, *P*=0.0003). The overall survival rate of the patients with flTF and asTF either high group was significantly better than those with flTF and asTF both high group (Figure 3E, *P*=0.012). We also found there was a significant difference among the three subgroups, namely the patients with flTF and asTF both low, the patients with flTF and asTF either high, and the patients with flTF and asTF both high (Figure 3F, *P*=0.001).

**Table 2:** Cox model analysis of flTF mRNA expression level in predicting gastric patients’ prognoses

Clinical parameters	Univariate			Multivariate		
	HR	95% CI	*P-*value	HR	95% CI	*P-*value
Gender						
Male / Female	0.76	0.31∼1.84	0.543	1.36	0.27∼6.74	0.707
Age (year)						
50-60/<50	0.37	0.11∼1.23	0.104	2.63	0.32∼21.45	0.366
≥60/<50	0.26	0.09∼0.76	0.014*	2.13	0.19-24.28	0.543
Histo-pathological type						
Ulcer / Invasive	0.54	0.21∼1.40	0.204	1.34	0.25∼7.19	0.733
Others / Invasive	0.72	0.22∼2.37	0.594	6.80	0.71∼65.52	0.097
Tumor size						
≥5cm/<5cm	7.03	1.62∼30.54	0.009*	13.98	1.95∼100.34	0.009*
Depth of invasion						
Whole layer / Non-whole layer	1.88	0.62∼5.72	0.268	0.87	0.12∼6.10	0.885
Nodal metastasis						
Yes / No	1.49	0.35∼6.38	0.595	4.05	0.47∼34.71	0.202
Differentiation						
Poor / Well	2.33	0.91∼5.94	0.077	3.38	0.92∼12.44	0.067
Distant metastasis						
Yes / No	3.04	0.97∼9.52	0.056	16.57	2.10∼130.48	0.008*
flTF mRNA expression level						
High / Low	3.19	1.22∼8.35	0.018*	6.03	1.02∼35.71	0.048*

Values in bold signify *P* < 0.05.

**Table 3:** Cox model analysis of asTF mRNA expression level in predicting gastric patients’ prognoses

Clinical parameters	Univariate			Multivariate		
	HR	95% CI	*P-*value	HR	95% CI	*P-*value
Gender						
Male / Female	0.76	0.31∼1.84	0.543	0.52	0.07∼4.13	0.534
Age (year)						
50-60/<50	0.37	0.11∼1.23	0.104	7.45	0.52∼106.87	0.140
≥60/<50	0.26	0.09∼0.76	0.014*	2.63	0.25∼27.44	0.419
Histo-pathological type						
Ulcer / Invasive	0.54	0.21∼1.40	0.204	1.98	0.39∼10.19	0.414
Others / Invasive	0.72	0.22∼2.37	0.594	25.91	2.02∼331.81	0.012*
Tumor size						
≥5cm/<5cm	7.03	1.62∼30.54	0.009*	28.56	3.50∼233.00	**0.002** *
Depth of invasion						
Whole layer / Non-whole layer	1.88	0.62∼5.72	0.268	3.10	0.581∼16.54	0.185
Nodal metastasis						
Yes / No	1.49	0.35∼6.38	0.595	4.12	0.40∼42.48	0.235
Differentiation						
Poor / Well	2.33	0.91∼5.94	0.077	4.78	1.07∼21.33	0.041*
Distant metastasis						
Yes / No	3.04	0.97∼9.52	0.056	17.40	2.37∼127.85	0.005*
asTF mRNA expression level						
High / Low	2.50	1.05∼5.95	0.038*	10.74	2.32∼49.59	0.002*

Values in bold signify *P* < 0.05.

## DISCUSSION

TF is an initiation factor regulating extrinsic coagulation and multiple physiological and pathological processes such as tumor angiogenesis, wound healing, intracellular signaling, and tumor metastasis [4]. It has been demonstrated that high TF expression could be found in many different tumor cells, such as pancreatic carcinoma, lung carcinoma, and colorectal carcinoma, as well as in vascular endothelial cell and interstitial macrophages of tumor tissues [13]. In addition, abnormal expression of TF is associated with cell growth, tumor invasiveness and metastasis, prognosis, and multi-drug resistance [14].

The open reading frame of human *TF* gene encodes 6 exons, with flTF containing all 6 exons. In 2003, a study by Bogdanov reported an alternatively spliced tissue factor (asTF) transcript in myeloid leukemia HL-60 cell through by selectively removing exon 5 [10]. In contrast to the flTF, asTF lacks a trans-membrane domain and contain unique amino acid sequence [10]. It has been demonstrated that asTF does not play a significant role during coagulation due to the truncation of the extracellular domain encoded by exon 5, but contributes to tumor-associated angiogenesis and cancer growth [11, 15–17]. A previous study suggested a paradigm for the role of flTF by interacting with FVIIa to affect angiogenesis through its cytoplasmic domain and PAR-2 activation [13]. However, unlike flTF, asTF does not affect angiogenesis via PAR-dependent pathways but relies on integrin ligation [18]. Currently, the role of *flTF* and *asTF* levels in human cancers remains elusive. Rollin *et al*. [19] demonstrated that the transcript level of *asTF* was an independent prognostic marker in NSCLC. Our data showed that both flTF and asTF were over-expressed on gastric cancer tissues, and their expression significantly associated with prognosis. We also analyzed the correlation between both flTF and asTF expression in gastric cancer and patients’ clinico-pathological characteristics such as gender, age, histologic grade, TNM stage, pathologic type. Except age, we found that flTF and asTF did not significantly associate with any clinical patient parameters.

Pre-mRNA splicing is an essential, ubiquitous, and precisely regulated process that occurs following gene transcription and before mRNA translation [20]. Alternative splicing can generate a variety of different transcripts from a single gene. Alternative splicing represents an important molecular mechanism of gene regulation in a series of physiological processes, including developmental programming, disease, and even cancer [21]. Many tumor-associated splice variants have clear diagnostic value and may serve as potential drug targets [22]. Thus, understanding the process of aberrant splicing and the detailed characterization of the splice variants may improve our understanding of malignant transformation targeting TF involved in gastric cancer progression and metastasis.

In this study, we established a set of self-designed *asTF* primers and TaqMan probe to detect *asTF* transcripts (Table 4). Our self-designed *asTF* forward primer is located at the junction of exon 4 and exon 6 of *TF*. As demonstrated in the previous literature [23], the *asTF* forward and reverse primers were located at the exon 4 and exon 6, respectively, and the *asTF* TaqMan probe was located at the junction of exon 4 and exon 6 of *TF*. According to this method, the pair of primers of *asTF* can amplify a special fragment of *asTF*, while it can also amplify the fragment of *flTF* containing exon 5. This suggests a primer competition leading to a decrease of the amplification efficiency of this pair of primers. Herein, we selected the gastric cancer tissues, used the *asTF* primers and TaqMan probes designed by ourselves as well as adopted from the literature respectively, for the real-time PCR assay. The PCR products were then determined in the 2% agarose electrophoresis (as shown in Figure 1), indicating an optimal system for our self-designed primers and TaqMan probe to detect *asTF* transcripts.

**Table 4:** Sequences of primers and probes

Genes	Sequences (5′→3′)
flTF (ref. [14])	
Forward primer	TGATGTGGATAAAGGAGAAAACTACTG
Reverse primer	CTACCGGGCTGTCTGTACTCTTC
Probe	FAM-TTCAAGCAGTGATTCCCTCCCGAACA-TAMRA
asTF (ref. [14])	
Forward primer	GGGATGTTTTTGGCAAGGACTTA
Reverse primer	CCAGGATGATGACAAGGATGATG
Probe	FAM-AATCTTCAAGTTCAGGAAAGAAATATTCTACATCATTGGA-TAMRA
asTF(self-designed)	
Forward primer	ATCTTCAAGTTCAGGAAAGAAATATTCTAC
Reverse primer	GCTCTGCCCCACTCCTGCC
Probe	FAM-TTGGAGCTGTGGTATTTGTGGTCATCATC-TAMRA
β-actin	
Forward primer	GGAAGGTGAAGGTCGGAGTC
Reverse primer	CGTTCTCAGCCTTGACGGT
Probe	FAM-TTTGGTCGTATTGGGCGCCTG-TANRA

In the present study, the multi-factor COX model was used to analyze the prognostic value of flTF and asTF expression in gastric cancer patients. The COX model included the age, gender, stage, histological grade, TNM stage, pathologic type, and flTF/asTF mRNA expression level. Our study showed that both flTF and asTF mRNA expression levels could be used as independent prognostic factors, supporting the notion that flTF and asTF levels in gastric cancer have important prognosis values.

## MATERIALS AND METHODS

### Patients and tissue samples

Gastric cancer tissues were collected from 52 patients who underwent surgical resection between January 2001 and April 2015 at our hospital (34 men and 18 women; median age at diagnosis, 58 years). No patients received pre-operative chemotherapy or radiotherapy. All gastric cancer tissues were confirmed as gastric adenocarcinoma by hematoxylin and eosin (H&E) staining after surgical resection, and cancer stages were assigned according to criteria established by the American Joint Committee on Cancer [24]. Moreover, 10 cases of adjacent normal tissues were analyzed as control samples. Detailed patient clinical parameters are shown in Table 1. The study protocol was approved by the ethics committee of the hospital.

### RNA isolation and reverse transcription

Total RNA in gastric tissues was extracted by using a total RNA purification kit (Biocolor BioScience and Technology Company, Shanghai, China) following the manufacturer's protocol. The quality of the RNA samples was determined by absorbance measurements at 260/280 nm. Two μg of total RNA was reverse transcribed to cDNA using the first strand cDNA synthetic kit (Fermantas, Vilnius, Lithuania) according to the manufacturer's instructions. cDNA standards were amplified by conventional PCR and the PCR product was purified by agarose-gel electrophoresis. DNA standards were extracted using the EZ-10 spin column DNA gel extraction kit (Sangon Biological Engineer Technology and Services Limited Corporation, Shanghai, China).

### Real-time PCR

All real-time PCR reactions were performed in the ABI 7500 (Applied Biosystem, USA) using a final volume of 20μl. Reactions were performed with 10μl of 2x TaqMan^®^ Universal PCR Master Mix PCR buffer, 2μl of 5μM forward primer, 2μl of 5μM reverse primer, 2μl of 2.5μM TaqMan probe, 2μl template cDNA, and 2μl ddH_2_O. The primers and TaqMan probes used to identify *flTF* were adapted from Szotowki *et al*. [23]. The primers and TaqMan probes of *asTF* and the reference gene *GAPDH* were designed according to the National Center for Biotechnology Information (NCBI) database by using the Primer Primier 5.0 software (Palo Alto, CA, USA). The forward primer of asTF was located at the junction of exon 4 and exon 6, which is different from the design of the reference [23]. The sequences of all primers and TaqMan probes used in the present study were listed in Table 4. The cycling conditions for flTF and asTF were as follows: pre-denaturation at 50°C for 2 min, initial denaturation at 95°C for 10 min, followed by 40 cycles at 95°C for 15 sec and 60°C for 1 min, collecting the fluorescence signal at 60°C. The data were normalized to GAPDH, and relative expression was calculated by the 2^−ΔΔCT^ method.

### Statistical analyses

Statistical analyses were performed using the GraphPad Prism 4.0 software package (GraphPad Software, Inc., San Diego, USA). Paired Student's *t*-test, Wilcoxon signed rank test, or the log rank survival analysis were used where appropriate. A *p*-value of <0.05 was determined as statistically significant.
